# Overweight in Childhood, Adolescence and Adulthood and Cardiovascular Risk in Later Life: Pooled Analysis of Three British Birth Cohorts

**DOI:** 10.1371/journal.pone.0070684

**Published:** 2013-07-24

**Authors:** Min Hae Park, Ulla Sovio, Russell M. Viner, Rebecca J. Hardy, Sanjay Kinra

**Affiliations:** 1 Department of Non-communicable Disease Epidemiology, London School of Hygiene and Tropical Medicine, London, United Kingdom; 2 Department of General and Adolescent Paediatrics, UCL Institute of Child Health, London, United Kingdom; 3 MRC Unit for Lifelong Health and Ageing, Institute of Epidemiology and Health Care, University College London, London, United Kingdom; Tulane School of Public Health and Tropical Medicine, United States of America

## Abstract

**Background:**

Overweight and obesity in adulthood are established risk factors for adverse cardiovascular outcomes, but the contribution of overweight in childhood to later cardiovascular risk is less clear. Evidence for a direct effect of childhood overweight would highlight early life as an important target for cardiovascular disease prevention. The aim of this study was to assess whether overweight and obesity in childhood and adolescence contribute to excess cardiovascular risk in adults.

**Methods and findings:**

Data from three British birth cohorts, born in 1946, 1958 and 1970, were pooled for analysis (n = 11,447). Individuals were categorised, based on body mass index (BMI), as being of normal weight or overweight/obese in childhood, adolescence and adulthood. Eight patterns of overweight were defined according to weight status at these three stages. Logistic regression models were fitted to assess the associations of patterns of overweight with self-reported type 2 diabetes, hypertension, and coronary heart disease (CHD) in adulthood (34–53 years). Compared to cohort members who were never overweight, those who were obese in adulthood had increased risk of all outcomes. For type 2 diabetes, the odds ratio was higher for obese adults who were also overweight or obese in childhood and adolescence (OR 12.6; 95% CI 6.6 to 24.0) than for those who were obese in adulthood only (OR 5.5; 95% CI 3.4 to 8.8). There was no such effect of child or adolescent overweight on hypertension. For CHD, there was weak evidence of increased risk among those with overweight in childhood. The main limitations of this study concern the use of self-reported outcomes and the generalisability of findings to contemporary child populations.

**Conclusions:**

Type 2 diabetes and to a lesser extent CHD risk may be affected by overweight at all stages of life, while hypertension risk is associated more strongly with weight status in adulthood.

## Introduction

Overweight and obesity in adulthood are known risk factors for adverse cardiovascular outcomes including type 2 diabetes, hypertension, and coronary heart disease. [1] Reviews have shown that childhood obesity is also associated with cardiovascular outcomes [2], [3] and their risk factors, [4] but the relative contributions of overweight at different life-stages to cardiovascular risk is less clear. [5] The question of whether overweight in early life increases the risk of cardiovascular morbidity in obese adults has implications for approaches to intervention, as evidence for a direct effect of childhood overweight on these outcomes would highlight early life as a important target for obesity prevention and treatment.

Studies have attempted to address this issue by using standard adjustment for body size at different ages in regression analyses, but this approach can lead to overadjustment and artifactual associations. [6], [7] Alternative approaches to analysis that have been developed for testing life-course hypotheses can address some of these limitations. [8], [9] The aim of this study was to assess whether overweight and obesity in childhood and adolescence contribute to excess cardiovascular risk in adults, which was achieved through examination of the relationships between patterns of overweight over the life-course and type 2 diabetes, hypertension, and coronary heart disease.

## Methods

### Study Cohorts

The three British National Birth cohorts used in this study have been described in previous publications.[10]–[12] In brief, the Medical Research Council (MRC) National Survey of Health and Development (NSHD) followed up all singleton babies of women with husbands in non-manual or agricultural jobs and one in four babies of women with husbands in manual jobs, who were born in England, Wales and Scotland during one week in March 1946. Sample size at baseline was 5,362, and the cohort has been followed up on more than twenty occasions. The National Childhood Development Survey (NCDS) included all babies born in Britain during one week in March 1958, and immigrants to the country who were born in the same week were incorporated into the study at ages 7, 11 and 16 years. There were 17,638 participants at baseline, who have been followed up on nine occasions. The British Cohort Study 1970 (BCS70) followed up 16,567 babies born in Britain in one week in April 1970. Immigrants were incorporated at ages 5, 10 and 16 years, and the cohort has been followed up on eight occasions. NSHD data were obtained from the MRC NSHD team at University College London. Data for NCDS [13]–[15] and BCS70 [16]–[18] were obtained from the UK Data Archive through the Economic and Social Data Service.

The present study included participants from the three cohorts who were singleton births, had height and weight (for body mass index BMI, kg/m^2^) measured in childhood and adolescence, and information on height and weight and the disease outcomes of interest in adulthood. Data collected at the following ages were used: in NSHD, BMI at ages 7, 15 and 43 years, and disease outcomes measured at age 53 years; in NCDS, BMI at ages 7, 16 and 42, and outcomes at age 46 years; and, in BCS70, BMI at ages 10, 16 and 34, and outcomes at age 34.

### Study Outcomes

The outcomes were self-reported type 2 diabetes, hypertension, and coronary heart disease (CHD). Participants were classified as having a disease outcome of interest if they reported ever having had the disease or treatment for that disease, in response to questions about the outcome, e.g. “Have you ever had diabetes?” These responses were combined with information on self-reported long-standing illnesses, reasons for medical supervision, or recent hospital admission, which were coded according to International Classification of Diseases (ICD) codes. [19] Individuals with any of the disease outcomes of interest before age 20 years, or relevant congenital defects, were excluded from analyses of that outcome to account for pre-existing conditions.

### Exposures

Height and weight in childhood and adolescence were measured in clinical examinations. At each examination, height and weight were measured once by a doctor or nurse. Adult height and weight were measured in NSHD, and self-reported in NCDS and BCS70. Overweight and obesity in childhood and adolescence were defined using international age- and sex-specific BMI centiles, and cut-off points corresponding to definitions of overweight (BMI 25 kg/m^2^) and obesity (BMI 30 kg/m^2^) in adulthood. [20], [21] Using these definitions, participants were categorised into one of eight groups according to overweight status in childhood and adolescence (not overweight versus overweight or obese) and obesity in adulthood (not obese versus obese). For example, group 1 (never overweight) included those who were not overweight or obese in childhood and adolescence, and not obese in adulthood, and group 8 (persistent overweight) included those who were overweight or obese in childhood and adolescence, and obese in adulthood. Overweight was selected in childhood and adolescence while obesity was used in adulthood due to the small number of obese individuals in earlier life and few normal weight cohort members in adulthood.

Individuals were also categorised according to the sensitive periods at which they were exposed to overweight or obesity (childhood, adolescence or adulthood), and the number of life stages at which they were overweight (one, two or three time points). The sensitive period model, [22] in which exposure to overweight in certain windows during the life course has a greater impact on later health than exposure in other periods, was examined by comparing disease risk in individuals with overweight at each of these sensitive periods against disease risk in those with exposure to overweight at other points in the life course, e.g. for childhood as a sensitive period, disease risk among those overweight in childhood (regardless of weight status in adolescence and adulthood) was compared to disease risk among those who were overweight in adolescence and/or adulthood, but not in childhood. The accumulation of risk model, [22] in which the effects of exposure to overweight over the life-course accumulate to determine overall disease risk, were examined by comparing disease risk in individuals with different numbers of life stages of exposure to overweight.

### Statistical Analysis

Individual level data from the three cohorts were pooled for the main analyses. The characteristics of the study population were described using means and standard deviations for continuous variables (age and BMI), and frequency and percentages for categorical variables. Logistic regression models were fitted to assess the associations between different overweight patterns and each cardiovascular outcome. Results were presented as odds ratios (OR) and 95% confidence intervals (CI) for each overweight pattern, and odds ratios for each overweight pattern were compared in a pairwise fashion using an adjusted Wald test. Logistic regression models were also used to assess associations between overweight in each potential sensitive period and the accumulation score and cardiovascular outcomes. All analyses were adjusted for sex, year of birth (cohort, as a categorical variable), age at measurement of childhood weight and height, birth weight, socioeconomic position (SEP) at birth based on the occupation of head of household, SEP in adulthood based on current occupation, and smoking status in adulthood. Analyses for CHD risk were additionally adjusted for hypertension and type 2 diabetes. Sample weights were applied to the dataset to account for the weighted sample design in NSHD. Modification of the main effect of patterns of overweight by sex and by cohort was assessed by the inclusion of interaction terms. All statistical analyses were carried out using Stata SE, version 12.1 (StataCorp, TX, USA).

## Results

The characteristics of study participants in each cohort are presented in Table 1. Across all three cohorts, the prevalence of overweight or obesity in childhood was 7.8% and the prevalence of obesity was 1.0%. These figures were 11.1% and 1.7%, respectively in adolescence, and 54.3% and 17.2% in adulthood.

**Table 1:** Characteristics of participants in three British birth cohorts (born 1946, 1958 and 1970) with height and weight data measured in childhood, adolescence and adulthood, by cohort.

Variable	Cohort n (%) or mean (SD)		
	1946	1958	1970
	N = 2,211	N = 6,207	N = 3,029
**Sex**			
Male	1,122 (50.8)	3,102 (50.0)	1,381 (45.6)
Female	1,089 (49.3)	3,105 (50.0)	1,648 (54.4)
**Ethnicity**			
White	n/a*	6,133 (98.8)	2,971 (98.1)
Non-white		74 (1.2)	68 (1.9)
**Child characteristics**			
Age	7.1 (0.20)	7.3 (0.25)	10.2 (0.20)
BMI	15.8 (1.41)	15.9 (1.69)	16.9 (2.10)
Overweight and obese	155 (7.6)	515 (9.3)	261 (8.6)
Obese	14 (0.6)	87 (1.4)	9 (0.3)
**Adolescent characteristics**			
Age	14.5 (0.20)	15.8 (0.19)	16.4 (0.25)
BMI	20.0 (2.67)	20.7 (2.85)	21.2 (3.12)
Overweight and obese	195 (8.8)	622 (10.0)	395 (13.0)
Obese	17 (0.8)	95 (1.5)	57 (1.9)
**Adult characteristics**			
BMI	25.5 (4.25)	26.7 (4.96)	25.7 (4.77)
Overweight and obese	1,072 (48.5)	3,703 (59.7)	1,469 (48.5)
Obese	281 (12.7)	1,155 (18.6)	492 (16.2)
Hypertension	1,288 (58.2)	692 (13.5)	140 (4.6)
Type 2 diabetes	59 (2.7)	135 (2.6)	33 (1.1)
Coronary heart disease	85 (4.4)	18 (0.4)	2 (0.1)

* Ethnicity data were not available for these analyses; for the purposes of consequent analyses, cohort members were assumed to be of white ethnicity. BMI – body mass index; SD – standard deviation.

Among the 11,447 cohort members with height and weight data at all three life stages, the majority (75%) were never overweight (neither overweight nor obese in childhood and adolescence, and not obese in adulthood). Upwards movement in weight categories with age was relatively common, with 10% of cohort members being normal weight in childhood and adolescence and obese in adulthood, and a further 3.4% becoming overweight or obese in adolescence and remaining obese in adulthood. 2.3% of cohort members were persistently overweight or obese at all three life stages. It was uncommon for cohort members who had been overweight in childhood and adolescence to become non-obese adults (1.4%). Cohort members who were overweight at more life periods had higher BMI in adulthood. Those who were overweight at a single life stage had mean BMI 30.3 kg/m^2^ (standard deviation SD 5.36), compared to 32.8 (SD 5.51) among those overweight at two stages, and 36.4 (SD 4.82) among those with persistent overweight.

Associations between the different patterns of overweight and cardiovascular outcomes are presented in Table 2. Interaction terms for sex and cohort were not statistically significant at conventional levels (*P*<0.05), therefore results are presented for all cohort members combined.

**Table 2:** Associations between patterns of overweight in childhood, adolescence and adulthood and cardiovascular outcomes in three British birth cohorts, from logistic regression analyses.

Overweight pattern	Weight status*			n	Adult BMI Mean (SD)	Hypertension OR (95% CI)	Type 2 diabetes OR (95% CI)	CHD OR (95% CI)
	Childhood	Adolescence	Adulthood					
Never overweight	0	0	0	8,587	24.4 (2.79)	1	1	1
Childhood only	1	0	0	374	25.3 (2.53)	0.87 (0.54 to 1.40)	0.99 (0.35 to 2.80)	0.44 (0.20 to 1.89)
Adolescence only	0	1	0	397	26.5 (2.38)	0.97 (0.61 to 1.55)	0.88 (0.31 to 2.50)	1.63 (0.37 to 7.19)
Adulthood only	0	0	1	1,144	33.3 (4.64)	2.28 (1.76 to 2.95)	5.47 (3.39 to 8.82)	3.83(1.98 to 7.42)
Childhood+adolescence	1	1	0	161	26.4 (2.20)	1.01 (0.46 to 2.21)	1.24 (0.29 to 5.25)	3.43 (0.60 to 19.64)
Childhood+adulthood	1	0	1	130	34.0 (4.78)	2.91 (1.54 to 5.49)	4.70 (1.89 to 11.67)	1.10 (0.14 to 8.48)
Adolescence+adulthood	0	1	1	388	35.0 (4.61)	3.01 (2.11 to 4.29)	6.61 (3.61 to 12.09)	3.74(1.35 to 10.35)
Persistent overweight†	1	1	1	266	36.4 (4.82)	2.56 (1.40 to 4.68)	12.60 (6.61 to 23.98)	6.62(1.94 to 22.65)

* In childhood and adolescence, 0 = normal weight; 1 = overweight or obese. In adulthood, 0 = nonobese; 1 = obese.

† Persistent overweight: overweight in childhood and adolescence and obese adulthood. OR is for odds of disease outcome compared to never overweight category; Adjusted for sex, year of birth, exact age and height at childhood BMI measurement, birth weight, SEP at birth SEP in adulthood, and smoking status in adulthood; adjusted for weighted sampling in NSHD.

### Type 2 Diabetes

Obesity in adulthood in combination with any pattern of overweight in earlier life was associated with increased odds of type 2 diabetes, compared to never overweight. ORs for type 2 diabetes were higher for persistent overweight (OR 12.6; 95% CI: 6.6, 24.0) than for obesity in adulthood only (OR 5.5; 95% CI: 3.4, 8.8) (*P*-value from adjusted Wald test = 0.015). Compared to those who were never overweight, cohort members with overweight isolated to childhood or adolescence did not have increased odds of type 2 diabetes. Sensitive period effects of overweight in childhood or adolescence were not observed, but obesity in adulthood was associated with higher odds compared to exposure in other periods (*P*-value from adjusted Wald test <0.001). Examination of the ORs from the accumulation of risk model indicated increasing risk of type 2 diabetes with overweight at more life stages (*P*-value from test for trend = 0.018).

### Hypertension

Obesity in adulthood in combination with any other pattern of overweight in childhood and adolescence was associated with increased odds of hypertension compared to a pattern of never overweight. The OR for persistent overweight (OR 2.56; 95% CI: 1.40, 4.68) was not different to that for obesity in adulthood only (OR 2.28; 95% CI: 1.76, 2.95). Overweight isolated to childhood or adolescence was not associated with increased odds of the outcome. There was no indication of a sensitive period effect of overweight in childhood or adolescence, but there was evidence for an effect of obesity in adulthood (*P*<0.001). There was no evidence for a cumulative effect of overweight over the three time periods on hypertension risk (*P* = 0.127).

### Coronary Heart Disease

Compared to a pattern of never overweight, obesity in adulthood only, overweight in adolescence and adulthood, and persistent overweight were associated with increased odds of CHD. The OR for persistent overweight was not statistically different to the OR for obesity in adulthood only at conventional levels of statistical significance (*P*<0.05), but examination of point estimates suggested higher odds associated with persistent overweight (OR for persistent overweight 6.62, versus 3.83 for obesity in adulthood only). This difference was attenuated after adjustment for adult BMI. When the model was adjusted for hypertension, there was little change in the effect sizes. On adjustment for type 2 diabetes, effect sizes for obesity in adulthood only and persistent overweight were attenuated (OR for obesity in adulthood decreased from 3.83 to 2.94, and OR for persistent overweight decreased from 6.62 to 4.84). There was no evidence for a sensitive period effect of overweight and obesity at any of the three time periods, or an accumulation of risk effect on CHD.

## Discussion

This study has shown that overweight in childhood and adolescence is associated with increased risk of type 2 diabetes among obese adults. In contrast, an effect of overweight in childhood and adolescence on hypertension risk was not observed.

Compared to never being overweight, persistent overweight through childhood, adolescence and adulthood was associated with a 12-fold increase in odds of type 2 diabetes, while obesity in adulthood only was associated with a five-fold increase. This finding is in contrast to a previous study, which found no difference in the relative risks of type 2 diabetes between those with persistent overweight and those with obesity isolated to adulthood [23]; however, the outcomes were reported at a younger age than in the present study (mean age 31–39 years) and there were fewer cases of type 2 diabetes, which may have reduced the power to detect a difference. The present study showed evidence of increasing type 2 diabetes risk with overweight at more life stages, but an effect of overweight in early life was not observed in cohort members that were not obese in adulthood. A conceptualisation of disease progression in which overweight contributes to increasing insulin resistance and loss of beta cell function over time, [24] eventually leading to type 2 diabetes, [25] would be consistent with these observations. Individuals that are exposed to overweight in childhood and become non-obese in adulthood may have raised levels of insulin resistance and glucose, which do not reach the threshold for a diagnosis of type 2 diabetes when levels of overweight in adulthood are reduced. Persistent overweight could also indicate some underlying genetic predisposition to overweight and metabolic complications. [26] This model highlights the potential benefits of early intervention to reduce overweight and minimise individuals’ exposure to insulin resistance. Beneficial effects of weight loss on indices of insulin sensitivity have been demonstrated in obese children and adolescents. [27], [28].

In contrast to the findings for type 2 diabetes, exposure to overweight in early life did not increase the risk of hypertension in obese adults. These associations appear to be more consistent with a model in which obesity in adulthood accounts predominantly for hypertension risk, rather than longer term effects associated with overweight in childhood and adolescence. This is unlike a previous study, which showed that overweight in childhood and adolescence was associated with additional hypertension risk compared to obesity in adulthood alone. [23] However, other studies have shown that lower BMI in childhood in combination with overweight in adulthood is associated with the highest risk of hypertension, [4] indicating that rapid growth over the life-course may be important for determining hypertension risk. [29], [30] An alternative explanation could be that obesity in adulthood is a marker for other risk factors, which may act over the life-course or more contemporaneously in adulthood, to determine hypertension risk. For example, body size in adulthood may be linked to aspects of diet and physical activity that are associated with hypertension, or to factors that act on hypertension risk through psychosocial pathways, such as anxiety and depression. [31], [32] BMI in later adulthood may also be a good indicator of cumulative exposure to overweight in adulthood. [33] The mechanisms by which obesity causes hypertension are not well understood, [34] and there may be multiple pathways to elevated blood pressure; further examination of the relative contributions of overweight and other risk factors to hypertension risk will identify where intervention efforts should be focused.

Point estimates for CHD indicated that there may be higher risk associated with persistent overweight. The relatively young ages at measurement leading to small number of CHD cases meant that the study may have been underpowered to detect a difference, therefore studies with larger sample sizes and/or follow-up to older ages may be warranted. Given that elevated blood sugar and high blood pressure are known risk factors for cardiovascular disease, [35], [36] the relationship between overweight and CHD may be driven in part by the associations of overweight with these two conditions; for example, a large study of data from 58 prospective studies indicated that BMI in adulthood did not improve prediction of cardiovascular disease risk when information on systolic blood pressure, diabetes history and serum lipids was available. [37] The contrasting relationships between these cardiovascular risk factors and overweight over the life-course may explain the intermediate effect on CHD that has been observed, with overweight acting on type 2 diabetes risk from early life and on hypertension at later ages. A model in which childhood overweight contributes to type 2 diabetes risk, and hypertension and obesity in later life contribute to CHD risk may be proposed as a possible framework for understanding cardiovascular risk. Disentangling the interrelationships between overweight and other cardiovascular risk factors, and their contributions to type 2 diabetes, hypertension and CHD over the life-course will be key for understanding the mechanisms to cardiovascular disease, and for determining the timing and nature of interventions to reduce cardiovascular burden.

Limitations of this study include the use of self-reported outcomes which may be subject to measurement error and biases, [38] and the small number of cases of cardiovascular disease outcomes. Another limitation is the use of BMI measurements at single ages to represent adiposity during a period of development; these measurements do not reflect the changes in body size and composition that take place over the life-course, [39], [40] and which may be associated with cardiovascular outcomes. [41], [42] Analysis of overweight and obesity in early life as separate categories was limited by the low prevalence of childhood obesity in these cohorts, and combining these in a single exposure category may have masked the effects of more extreme overweight in youth. A more general limitation of cohort studies with long-term follow-up concerns the generalisability of findings to contemporary populations of children; today’s child population is experiencing more extreme obesity [43] and at earlier ages [44] than has previously been observed, and the effects on long-term health may be different to those observed in older cohorts. However, in the absence of more robust study designs, data from observational studies can be used to provide insight into these relationships. Due to the small proportion of ethnic minority groups in the study sample (∼1%), analyses were not stratified by ethnicity. However, it is known that obesity and cardiovascular risk vary by ethnic group, with ethnic minority groups in the UK generally experiencing higher prevalence of both obesity and cardiovascular diseases than White ethnic groups. [45], [46] Consequently the findings of this study cannot be generalised to ethnic minority populations in the UK, for which cardiovascular risk and its relationship with weight status may be different.

This analysis of pooled data from three nationally representative cohort studies showed that overweight in childhood and adolescence was associated with increased risk of type 2 diabetes, but not hypertension in obese adults. Type 2 diabetes may have a different relationship to overweight over the life-course than hypertension. Research that examines the relationships between the various cardiovascular risk factors and their evolution over the life-course will be important for understanding the pathways that lead to cardiovascular disease, and may help to determine the preferred timing and nature of interventions to prevent cardiovascular diseases.
